# Preparation of the Nanostructured Ni-Mg-O Oxide System by a Sol–Gel Technique at Varied pH

**DOI:** 10.3390/nano12060952

**Published:** 2022-03-14

**Authors:** Grigory B. Veselov, Timofey M. Karnaukhov, Vladimir O. Stoyanovskii, Aleksey A. Vedyagin

**Affiliations:** 1 Department of Materials Science and Functional Materials, Boreskov Institute of Catalysis SB RAS, 630090 Novosibirsk, Russia; g.veselov@catalysis.ru (G.B.V.); karnaukhovtm@catalysis.ru (T.M.K.); stoyn@catalysis.ru (V.O.S.); 2 Faculty of Natural Sciences, Novosibirsk State University, 630090 Novosibirsk, Russia

**Keywords:** nanostructured MgO, Ni-based system, sol–gel synthesis, thermal stability, chemical looping

## Abstract

In the present work, a series of two-component Ni-Mg-O oxide systems were prepared using a sol–gel technique at varied pH of hydrolysis procedure. The aqueous solutions of nitric acid or ammonia were added to control the pH values. The xerogel samples obtained after drying were analysed using a thermogravimetric approach. The oxide systems were characterized by a set of physicochemical methods (low-temperature nitrogen adsorption, X-ray diffraction analysis, scanning electron microscopy, UV-vis spectroscopy, and temperature-programmed reduction method). The thermal stability of the samples was examined in a testing reaction of CO oxidation in a prompt thermal aging regime. It was revealed that the pH value during the magnesium methoxide hydrolysis stage significantly affects the properties of the intermediate hydroxide and final oxide nanomaterials. The thermal decomposition of nitric acid or ammonia is accompanied by exothermal effects, which noticeably influence the textural characteristics. Moreover, the pH of the hydrolysing solution defines the strength of the nickel interaction with the MgO matrix. An increase in pH facilitates the formation of the Ni_x_Mg_1−x_O solid solution with a higher amount of incorporated nickel, which is characterized by the reproducible broad temperature range of the hydrogen uptake and the enhanced thermal stability.

## 1. Introduction

A set of unique properties of nickel oxide makes it attractive for a number of application areas. For instance, it is used as a component of batteries, fuel cells, supercapacitors and sensors [1,2,3,4,5]. Moreover, nickel oxide is considered for antimicrobial applications [6]. In catalysis and catalytic processes, NiO is also a very important compound. It serves as an active component of the catalysts for the Suzuki cross-coupling, thermal decomposition of nitrocellulose, photocatalytic decomposition of methylene orange and methylene blue, etc. [7,8,9]. Another potential application of nickel oxide is its usage as an oxygen storage material in chemical looping processes [10]. The principle of the chemical looping technology deals with the complete or partial oxidation of various substances, mainly hydrocarbons, by the transition metal oxide serving as an oxygen carrier. After this stage, the reactor with the deoxygenated material is purged with the air flow. This regeneration stage is required to restore the initial oxidation state of the transition metal and to remove the coke deposits, which could be formed due to the implementation of the side reactions. These two stages constitute the complete chemical cycle. Among the transition metals, oxides of nickel and cobalt are the most promising candidates to function as oxygen carriers. Thus, Mattisson et al. [11,12] have recently reported that alumina-supported NiO provides the complete combustion of methane within the temperature range of 700–1200 °C. The yield of CO_2_ formed from methane can reach 99.8%. Shen et al. [13] have studied the chemical looping combustion of coal using NiO and reported a carbon conversion efficiency of 92.8% in this process.

Among the methods to prepare the oxide systems with desired properties, the sol–gel approach gives the widest opportunities. The resulting materials possess a developed surface area and uniform structure and texture. It is important to note that this method is simple from a technological point of view. In the case of two-component systems, it provides an even distribution of one oxide within the matrix of another oxide. This was numerously reported for the compositions based on magnesium oxide [14,15,16,17].

For the sol–gel MgO-based systems, the most important preparation stage is the hydrolysis of magnesium methoxide. Usually, distilled water is used for this purpose [16,17]. In this case, the second component should be further introduced in the form of organometallic compounds [18], which are exotic and expensive precursors. An alternative approach has been reported in a number of recent publications [19,20,21,22,23]. In these papers, the hydrolysis stage was efficiently performed using an aqueous solution of the inorganic salt precursor. This allows introducing the second component simultaneously with the sol formation and, therefore, results in a very uniform distribution of this component within the MgO matrix. The two-component Ni-Mg-O system prepared via the sol–gel technique is characterized by high reproducibility in the reduction/oxidation cycles [21]. In the as-prepared system, Ni^2+^ ions substitute magnesium in the MgO lattice, thus forming the solid solution. During the first reduction cycle, this solid solution decomposes with the formation of dispersed Ni^0^ particles distributed within MgO and stabilized by this matrix. In the subsequent reduction/oxidation cycles, the system exhibits reproducible behaviour on hydrogen uptake in a temperature range of 350–550 °C.

At the same time, it is known that the preparation conditions can significantly affect the final properties of the oxide material. Thus, an addition of toluene is very important for the gel stabilization that affects the textural characteristics of the product [24]. Another influential factor is the water-to-magnesium methoxide ratio. On the other hand, the pH of the medium is proved to influence such processes taking place during the sol–gel synthesis as hydrolysis and polycondensation. For instance, in the case of silica preparation, the highest rate of silicon alkoxide hydrolysis is observed at the high and low pH values, while at neutral pH of 7, the rate is minimal [25]. For zinc oxide, an increase of pH from 3 to 11 was shown to enlarge the particles from 70 to 200 nm [26]. Titanium oxide, which is often used as a photocatalyst, can also be produced by sol–gel technology. In this case, the pH variations will affect the specific surface area, phase composition, and photocatalytic activity of the resulting TiO_2_ [27]. Ghorbani et al. [28] have reported that for the MgO–Y_2_O_3_ composite nanopowder obtained by the Pechini sol–gel method, a decrease in pH leads to smaller particle sizes and a higher surface area. The best textural characteristics were achieved when pH was 2, and the ratio of citric acid to yttrium was 10:1. The pH effect on the sol–gel synthesis of MgO was studied by Lopez and co-workers [29,30]. It is shown that at acidic pH, the preparation process is controlled by the hydrolysis of magnesium ethoxide, while at basic pH it is controlled by the polycondensation. The highest surface area was achieved for the sample prepared at pH = 9. It should be noted that in some cases the type of the used acid matters.

The present work aims to study the pH effect on the characteristics of two-component Ni-Mg-O. The hydrolysis of the magnesium methoxide was performed using an aqueous solution of nickel nitrate with varied pH. The decomposition of Ni-Mg-OH xerogel with the formation of Ni-Mg-O oxide was examined by the thermogravimetric analysis. The final oxide system was characterized by a set of methods (X-ray diffraction analysis, scanning electron microscopy, low-temperature nitrogen adsorption, UV-vis diffuse reflectance spectroscopy). The reduction/oxidation behaviour of the samples was studied in a temperature-programmed regime in nine consecutive cycles, and their thermal stability was estimated in a testing reaction of CO oxidation under the prompt thermal aging conditions.

## 2. Materials and Methods

### 2.1. Synthesis of the Ni-Mg-OH and Ni-Mg-O Samples

The first step of the preparation is the dissolution of the magnesium ribbon in methanol at continuous stirring with the formation of the saturated solution of magnesium methoxide. For each 1 g of magnesium, 43 mL of methanol was taken. Then, 43 mL of toluene was added to the solution as a gel stabilizer. Thus, the methanol-to-toluene ratio was 1:1. The next step: hydrolysis of magnesium methoxide, was performed dropwise using an aqueous solution of nickel nitrate instead of distilled water. This approach was recently reported as an effective route to prepare two-component systems [20]. It should be noted that the as-prepared solution of Ni(NO_3_)_2_ has a pH value of nearly 5. In order to vary the pH of the hydrolysing solution, nitric acid or ammonia were added. Thus, solutions with a pH of 1, 3, 7, and 9 were obtained.

Then, the formed gels were dried at room temperature for 2 h and in a furnace at 200 °C for another 2 h. The prepared xerogel samples were denoted as Ni-Mg-OH. In order to prepare the oxide samples, the xerogels were calcined at gradual heating to 500 °C with subsequent maintenance at this temperature for 1 h. The oxide samples were labelled as Ni-Mg-O. The content of NiO in all oxide samples was 15 wt%.

Two reference samples containing 2 and 5 wt% of NiO were additionally prepared by an incipient wetness impregnation method. The sample of pure MgO synthesized by the same procedures as described above. In this case, hydrolysis of magnesium methoxide was performed using distilled water. The calcined MgO sample was impregnated with an aqueous solution of nickel nitrate of desired concentration. Then, the samples were dried and calcined at 500 °C as described above.

### 2.2. Characterization of the Ni-Mg-OH and Ni-Mg-O Samples

The pH of the hydrolysing solution was monitored using a pH-meter Ohaus ST2100-F (Parsippany, NJ, USA).

Thermogravimetric (TG) and differential thermogravimetric (DTG) analyses were performed using a TG 209 F1 Iris thermobalance (NETZSCH-Gerätebau GmbH, Selb, Germany). The differential thermal analysis (DTA) was carried out using an STA 449F1 Jupiter analyser (NETZSCH-Gerätebau GmbH, Selb, Germany). The output gas mixture was analysed by a QMS 403D Aëolos quadrupole mass spectrometer (NETZSCH-Gerätebau GmbH, Selb, Germany). All measurements were made in a synthetic air (20 vol% O_2_ in Ar) fed at a gas flow rate of 60 mL/min. The temperature ramping rate was 10 °C/min.

The morphology and microstructure of the Ni-Mg-O samples were explored using a JSM-6460 (JEOL Ltd., Tokyo, Japan) scanning electron microscope (SEM).

The textural characteristics of the Ni-Mg-O samples (specific surface area, SSA; pore volume, V_pore_; average pore diameter, D_av_) were obtained by the low-temperature nitrogen adsorption/desorption measurements. The isotherms of nitrogen adsorption/desorption were recorded at 77 K using an ASAP-2400 (Micromeritics, Norcross, GA, USA) apparatus.

An X-ray diffraction (XRD) analysis of the samples was carried out using an X’TRA (Thermo ARL, Ecublens, Switzerland) diffractometer operating with a CuK_α_ radiation source in a 2θ angle range 15–85° with accumulation in each point of 5 s.

UV-vis diffuse reflectance spectra were registered in a range of 190 and 800 nm using a UV-vis spectrometer Varian Cary 300 UV/VIS Bio (Agilent Technologies, Palo Alto, CA, USA) with a DRA-CA-3300 integrating sphere (Labsphere, North Sutton, NH, USA). The Spectralon standard was used as a reference. The spectra were transformed into the Kubelka–Munk function F(R) [31]. The energy-gap width (E_g_) was estimated using the Tauc plot method for the direct allowed transitions.

The temperature-programmed experiments on reduction/oxidation cycling were carried out in a flow-through reactor system. The set-up allows regulating the gas flows and switching the reductive and oxidative gas mixtures in an automatic mode. The specimen (200 mg) was loaded inside the quartz reactor. The reactor was purged with a nitrogen flow of 40 mL/min for 30 min and then fed with the reductive gas mixture containing 10 vol% H_2_ in N_2_ (total flow rate of 45.7 mL/min). The reactor was heated from 30 to 700 °C with a ramping rate of 10 °C/min. The hydrogen concentration at the reactor outlet was measured using a hydrogen gas analyser GAMMA-100 (FSUE “SPA “Analitpribor”, Smolensk, Russia). The heated reactor was maintained at 700 °C for 15 min and cooled down to 30 °C in a nitrogen flow (40 mL/min). Then, the inlet gas mixture was switched to an air flow (10 mL/min), and the reactor was heated to 500 °C with a ramping rate of 20 °C/min. After maintaining at the final point for 30 min, the reactor was cooled down to 30 °C in an air flow, and the inlet gas mixture was switched back to the reductive mixture. The described procedures were repeated nine times.

The thermal stability of the samples was examined in a prompt thermal aging regime as described elsewhere [32,33]. Since nickel is known to be active in oxidation processes, the model reaction of CO oxidation was used as a catalytic response. The light-off temperature of this reaction depends on the dispersity of nickel species. Therefore, the shift in the light-off curves indicates the changes in nickel dispersity that can be due to agglomeration or re-dispersion processes. The prompt thermal aging regime considers an increase in the final temperature of the catalytic run after each second run. The experiments were arranged as follows. The specimen (300 mg) was loaded inside the quartz reactor. Then, the reactor was fed with the reaction mixture consisting of 1500 ppm of CO, 14.0 vol% of oxygen, and nitrogen as balance, with the flow rate of 334 mL/min. The reactor was heated from 50 °C with a ramping rate of 10 °C/min. The final temperature was 320 °C in cycles 1–2, 600 °C in cycles 3–4, 800 °C in cycles 5–6, 900 °C in cycles 7–8, and 1000 °C in cycles 9–11. The CO concentration at the reactor outlet was measured using a ULTRAMAT 6 gas analyser (Siemens, Munich, Germany). The temperature of 50% conversion of CO (T_50_) was the monitored parameter.

## 3. Results

**Figure 1:**
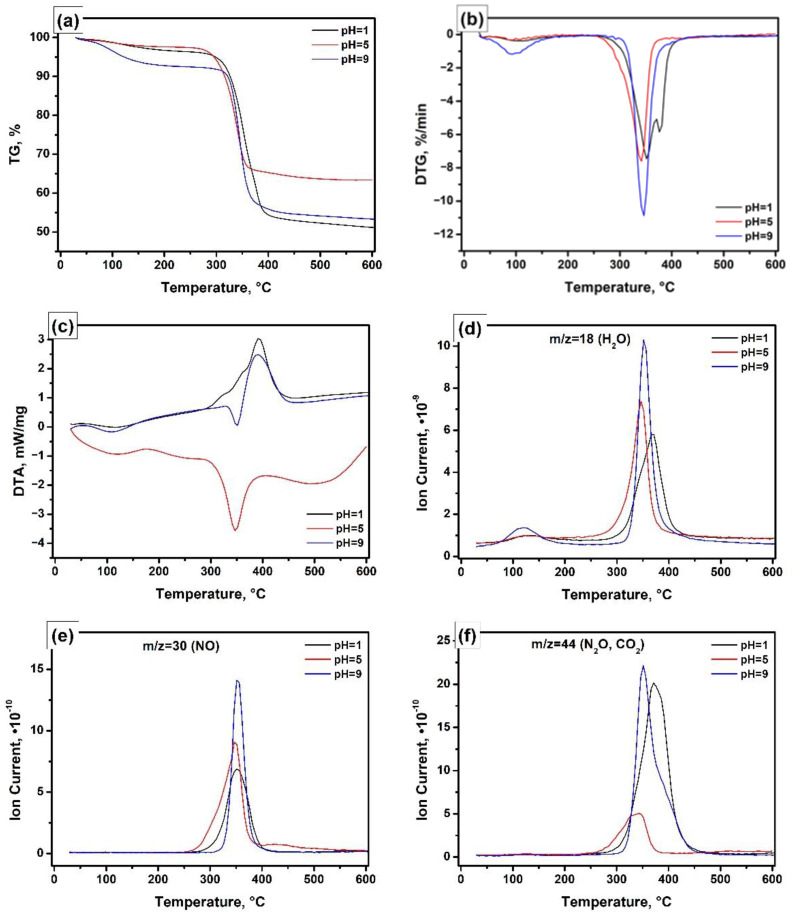
Thermal analysis of the Ni-Mg-OH samples prepared at various pH: (**a**) TG; (**b**) DTG; (**c**) DTA; (**d**–**f**) mass-spectrometry data.

The thermal decomposition behaviour of the Ni-Mg-OH xerogels prepared at various pH values was examined by thermal analysis techniques. Figure 1 compares the obtained results for the samples prepared at pH of 1, 5, and 9. As one can see from the thermogravimetric curves (Figure 1a), the total weight loss depends on the pH, since the samples contain additional amounts of nitric acid or ammonia species. Thus, when pH = 5, the observed weight loss was just 37%. The addition of ammonia to the solution to achieve pH = 9 leads to an increased weight loss of 47%. In the case of nitric acid (pH = 1), the weight loss reaches 49%. Figure 1b presents the differential curves. As follows from these curves, the character of the xerogel decomposition is also not the same. The sample Ni-Mg-O (pH = 5) decomposes in one step within a range of 255–370 °C with a maximum at 342 °C. The decomposition is accompanied by the endothermal effect (Figure 1c), which is typical for the magnesium hydroxide and nickel and magnesium nitrates [34,35,36,37]. According to the mass-spectrometry data (Figure 1d–f), the weight loss is mainly connected to the release of the hydrated water and the formation of NO/N_2_O species from the nickel nitrate. The low intensity of the fragments with *m*/*z* = 44 (Figure 1f) indicates that the formation of CO_2_ species is negligible. The DTG curve for the sample Ni-Mg-O (pH = 9) has two peaks (Figure 1b). As evident from Figure 1d, the low-temperature peak (47–153 °C with a maximum at ~90 °C) is assigned to the release of the chemically adsorbed water only. The second peak, narrow and intensive, is located in a range of 300–390 °C. For this peak, two effects, one endothermic at 350 °C and one exothermic at 390 °C, are observed in the DTA plot (Figure 1c). The first effect corresponds to the magnesium hydroxide and nickel nitrate decomposition described above. The appearance of the exothermic effect can be explained by the oxidation of ammonia species by nitrous groups [38]. This oxidation process significantly increases the amount of released water (Figure 1d). However, the high intensity of the fragments with *m*/*z* = 44 (Figure 1f) testifies towards the participation of residual organic species (methanol, toluene) in the oxidation process. The opposite sample, Ni-Mg-O (pH = 1), decomposes in a range of 270–410 °C with two peaks at 352 and 378 °C (Figure 1b). For these peaks, the DTA profile is represented by the exothermal effect only (Figure 1c). The corresponding exothermal peak has two left shoulders, which can be attributed to the superposition of one endothermal effect with another exothermal effect. The positions of the mass-spectrometric peaks in Figure 1d–f allow supposing that the invisible endothermal effect corresponds to the decomposition of nickel nitrate. In contrast, the intensive exothermal effect is related to the residual organic species oxidation catalysed by nitric acid [39,40]. Based on the described thermal analysis data, all xerogel samples were calcined at the temperature of 500 °C, which is enough to provide the complete decomposition of all precursors. In order to minimize the thermal effects, the temperature ramping rate within the range of 250–450 °C was as low as 1 °C/min.

**Figure 2:**
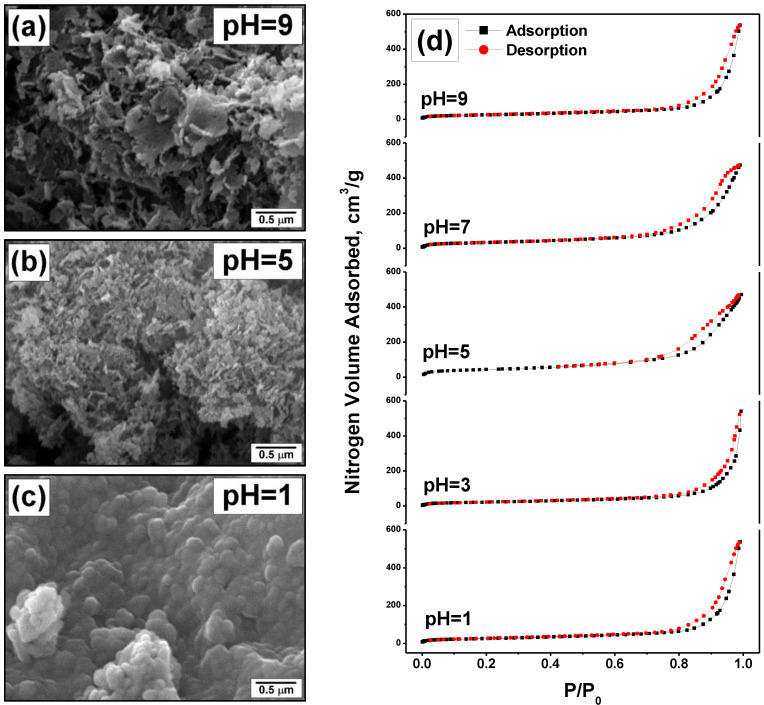
Characterization of the Ni-Mg-O samples obtained at various pH: (**a**–**c**) scanning electron microscopy images; (**d**) low-temperature nitrogen adsorption/desorption isotherms.

The oxide Ni-Mg-O samples after the calcination were characterized by scanning electron microscopy and a low-temperature nitrogen adsorption technique. The obtained results are shown in Figure 2 and Table 1. It is evident that the textural characteristics and morphology of the samples are influenced by the pH value during the hydrolysis procedure. Thus, the sample Ni-Mg-O (pH = 1) is composed of the relatively large primary particles agglomerated into a monolith-like sub-structure (Figure 2c). This sample possesses a smooth surface. At the same time, the pore volume and the average pore size for this sample are the largest ones (1.05 cm^3^/g and 41 nm, correspondingly). In the case of pH = 5, the resulting oxide sample has a fluffy morphology and is composed of the nanostructured primary particles (Figure 2b). All this explains the highest SSA value of 154 m^2^/g and the smallest pore size of 19 nm (Table 1). When the pH of the hydrolysing solution was 9, the prepared oxide sample exhibited the third type of morphology. The coral-like structure of this sample is shown in Figure 2a. It consists of more coarse particles forming the lacy 3D material. Along with this, in terms of textural parameters (SSA, pore volume, and average pore diameter), this sample is close to the sample Ni-Mg-O (pH = 1). The adsorption/desorption isotherms for all five samples are compared in Figure 2d. As one can note, all isotherms belong to Type IV with an H3 hysteresis loop. The shapes of the hysteresis loop for the samples Ni-Mg-O (pH = 5) and (pH = 7) are defined by the presence of micropores that makes them different from the other samples.

In general, the pH effects on the textural properties of Ni-Mg-O oxides can be summarized as follows. Firstly, the pH value, as well as the presence of additional ions in the hydrolysing solution, influences the process of the sol formation, thus defining the size of the further formed hydroxide particles. The higher pH values correspond to the smaller sizes of the primary particles. Secondly, these additional ions formed from ammonia or nitric acid significantly contribute to the thermal decomposition process, showing strong exothermal effect. At the same time, it is known that the temperature regime at the calcination stage is a texture-defining factor. Therefore, the exothermicity of the decomposition procedure results in the local overheating and gives trend to the sintering of the primary particles. This explains the microscopic images shown in Figure 2 and nitrogen adsorption data presented in Table 1. At pH = 1, the initially coarse particles become sintered into the large agglomerates. At the pH values of 5 and above, the primary particles are well dispersed. However, if ammonia is added, these fine particles also undergo sintering, thus forming the microstructured agglomerates (Figure 2a).

**Figure 3:**
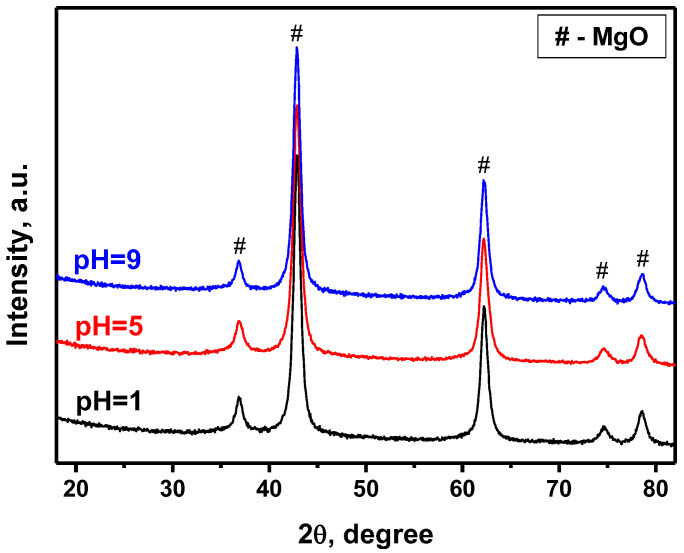
XRD patterns of the Ni-Mg-O samples obtained at various pH.

The characterization of the oxide Ni-Mg-O samples by X-ray diffraction analysis has revealed no noticeable difference between them in the phase composition (Figure 3). Due to the high dispersity of Ni species and their uniform distribution within the MgO matrix, these species are roentgen amorphous. The XRD patterns are represented by the reflections of the MgO phase only. The refining of the XRD data by the Rietveld method has revealed that the Mg atoms are partly substituted with the Ni atoms. The ionic radii of the Ni^2+^ and Mg^2+^ ions are close to each other (0.065 and 0.070 nm, correspondingly), and the MgO and NiO oxides possess the same *Fm3m* structure [41]. The lattice parameter of NiO is lower than that of MgO (Table 2). Therefore, the substitution of Mg^2+^ ions with Ni^2+^ ions should shift the reflexes towards higher angles. As one can see from Table 2, the observed effect is opposite. The lattice parameter is increased for all the MgO-based samples regardless of the nature of the second component. This is explained by the incorporation of the extraneous ions into the lattice of MgO, as well as by the small size of the MgO crystallites [16]. The joint effect of these two factors leads to the observed increase in the MgO lattice parameter to 4.219–4.220 Å. It should be noted that the indistinguishability of the NiO reflections in the XRD patterns of binary NiO-MgO systems was recently reported in the literature [41,42,43].

**Figure 4:**
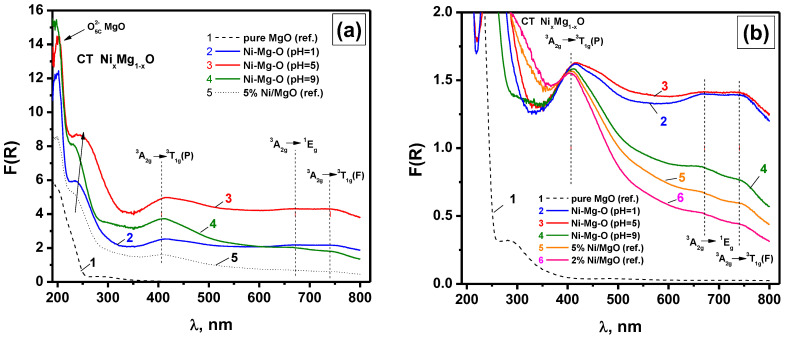
Initial (**a**) and normalized (**b**) UV-vis spectra of the Ni-Mg-O samples obtained at various pH and reference MgO and NiO/MgO samples.

However, the state and the dispersity of the nickel forms can be examined using UV-vis diffuse reflectance spectroscopy. Figure 4a demonstrates the UV-vis spectra for the Ni-Mg-O sample prepared at the pH of 1, 5, and 9. The spectrum of pure MgO synthesized by similar sol–gel procedures are given for comparison. Additionally, two more Ni-containing samples, 2%NiO/MgO and 5%NiO/MgO, were prepared by an incipient wetness impregnation of the sol–gel-prepared MgO with an aqueous solution of nickel nitrate with subsequent drying and calcination at 500 °C. These samples were also studied as references. The possible errors at the intensity measuring for the d-d bands can be related to the difference in the sizes of agglomerated particles. Therefore, in the present work, all the samples before recording the UV-vis diffuse reflectance spectra were thoroughly ground in an agate mortar to minimize such measurement errors. A more precise position of the d-d bands is seen in Figure 4b, where the spectra normalized in an area of the ^3^A_2g_ → ^3^T_1g_(P) band at 405 nm are presented.

The absorption spectra for the sol–gel-prepared Ni-Mg-O samples contain the band at 190–220 nm corresponding to O^2−^ on the MgO surface (surface five-fold coordinated anions, 6.6 eV, and four-fold coordinated anions on edge, 5.6 eV) [44]. The other part of the spectra is typical for Ni^2+^ ions in octahedral coordination and is characterized by the presence of the non-uniformly widened bands at 407, 671, and 741 nm. These bands are attributed to the most intensive transitions ^3^A_2g_ → ^3^T_1g_(P), ^3^A_2g_ → ^1^E_g_, and ^3^A_2g_ → ^3^T_1g_(F) [45]. It should be noted that according to the positions, these d-d transitions could be assigned to Ni^2+^ species in NiO, as well as in Ni_x_Mg_1−x_O, in a wide range of concentrations, including the trace amounts of Ni^2+^ in MgO [45]. The intensive absorption band of the charge transfer O^2−^(2p)→ Ni^2+^(3d) at 240–270 nm defines the energy-gap width E_g_ for the Ni_x_Mg_1−x_O particles [46]. The value of E_g_ depends on the Ni concentration. It was experimentally determined that E_g_ lies in an interval from 3.8 eV for NiO particles of ~6 nm in size to 7.8 for pure MgO [47]. It can be supposed that the size effects connected to an increase in the energy-gap width with the decrease in the particle size of NiO particles are lower than the effects related to the formation of solid solutions.

Thereby, the experimental spectra presented in Figure 4a for the Ni-Mg-O samples can be assigned to a mixed system consisting of both the Ni_x_Mg_1−x_O particles and Ni^2+^ ions in the MgO matrix. In order to estimate the contribution of each form, the impregnated samples NiO/MgO containing 2 and 5 wt% of NiO were prepared. For such reference samples calcined at 500 °C, the size of Ni_x_Mg_1−x_O particles are expected to be smaller, and the portion of Ni^2+^ ions in the MgO matrix should also be noticeably lower. As one can see from Figure 4b, the spectra 5 and 6 corresponding to the supported NiO/MgO sample are characterized by the relatively low intensity of the d-d transitions ^3^A_2g_ → ^1^E_g_ and ^3^A_2g_ → ^3^T_1g_(F) with regard to ^3^A_2g_ → ^3^T_1g_(P), if compared with the sol–gel-prepared samples Ni-Mg-O (pH = 1) and Ni-Mg-O (pH = 5). The intensities of the absorption bands in the area of four-fold and five-fold coordinated O^2−^ anions are increased for all samples in relation to pure MgO (Figure 4a). At the same time, in terms of the state of nickel species, the Ni-Mg-O (pH = 9) sample is closer to the supported samples. Note that for all the samples, the position of the d-d bands does not change.

**Figure 5:**
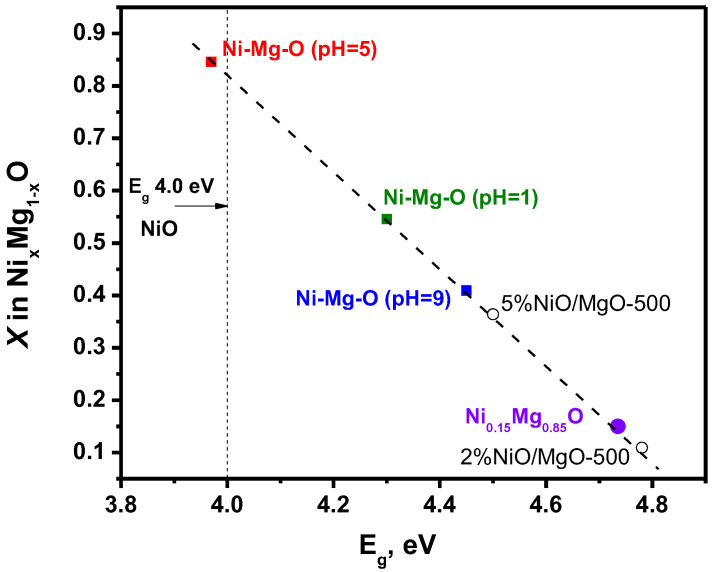
The values of the energy-gap width and corresponding Ni content estimated for the Ni_x_Mg_1−x_O particles.

For all Ni-Mg-O samples, the main changes in the UV-vis spectra consist of a rise in intensities of the d-d transitions and the charge transfer band in a row: pH = 1 < pH = 9 < pH = 5. Along with this, the edge of the charge transfer band shifts to the area of long waves, thus testifying the diminishing energy-gap width. However, as shown in Figure 5, the energy-gap width decreases in another order: pH = 9 > pH = 1 > pH = 5. According to the preformed estimation, the character value of E_g_ for the uniform distribution of Ni at x = 0.15 is found to be 4.7 eV [46]. Among the supported NiO/MgO samples, the closest value is observed for the sample containing 2% of NiO. This sample presumably contains the Ni_x_Mg_1−x_O particles of the smallest size. It is interesting to note that the decrease in the size of NiO particles supported on MgO results in their mutual interaction with the formation of an effective oxide cluster Ni_x_Mg_1−x_O with x~0.1. If the size of Ni_x_Mg_1−x_O particles increases, the effective portion of NiO species rises as well. The maximum content of Ni among the studied systems is found for the Ni-Mg-O (pH = 5) sample.

**Figure 6:**
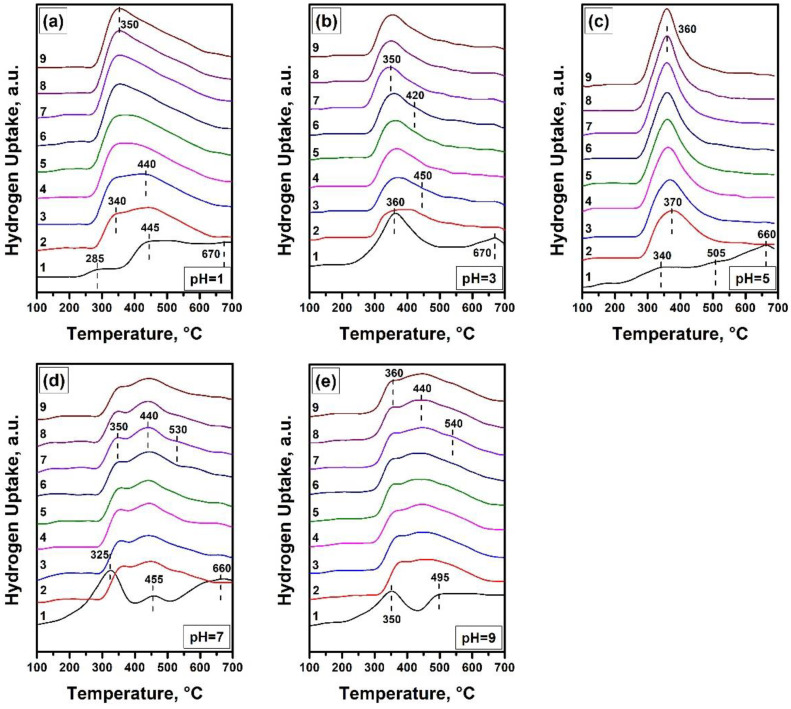
TPR profiles of the Ni-Mg-O samples obtained at various pH: (**a**) pH = 1; (**b**) pH = 3; (**c**) pH = 5; (**d**) pH = 7; (**e**) pH = 9.

The reduction/oxidation behaviour of the Ni-Mg-O samples was examined in nine consecutive cycles. The H_2_-TPR profiles of these cycles are shown in Figure 6. It is evident from the presented data that the pH significantly affects the redox properties of the nanomaterials. In all cases, the first reduction profile differs from the posterior ones. All materials start to exhibit reproducible behaviour after the third/fourth cycles only. Let us scrutinize each sample separately.

The first H_2_-TPR profile for the Ni-Mg-O (pH = 1) sample contains at least three hydrogen uptake peaks (Figure 6a). The peak at ~285 °C corresponds to the reduction in NiO particles distributed within the MgO matrix. The next peaks at ~445 and ~670 °C can be assigned to the decomposition of large and small particles of the Ni_x_Mg_1−x_O solid solution, accordingly. In the small particles, the interaction of NiO and MgO species is stronger than in the case of enlarged particles, and, therefore, their decomposition requires higher temperatures. For instance, it was numerously reported that the large NiO particles accessible for hydrogen are being reduced at lower temperatures [48,49,50,51]. Contrary, the small Ni-containing species penetrated in the structure of the NiO-MgO system undergo the reduction at significantly higher temperatures. After the first reduction/oxidation cycle, the increased number of NiO particles of larger size appear, thus shifting the first peak to ~340 °C. The Ni_x_Mg_1−x_O particles are also still present in the system. Due to their enlargement, the corresponding hydrogen uptake is observed as one peak at 440 °C. In the subsequent cycles, the first peak slightly shifts to ~350 °C and increases in intensity, while the second peak subsides and appears just as a shoulder.

The Ni-Mg-O (pH = 3) sample initially shows two hydrogen uptake peaks at ~360 °C and ~670 °C, corresponding to NiO and Ni_x_Mg_1−x_O particles (Figure 6b). The high intensity of the first peak indicates the presence of a large number of relatively coarse NiO particles. In the second and further cycles, the reduction profiles consist of two peaks: at ~360 °C slightly moving to 350 °C from cycle to cycle, and at 450 °C moving to 420 °C.

According to the UV-vis spectroscopy data, the Ni-Mg-O (pH = 5) sample is characterized by the largest Ni_x_Mg_1−x_O particles (Figure 5). As one can note from Figure 6c, the H_2_-TPR results confirm this fact. The first reduction in the sample proceeds through three steps: the reduction in a small amount of coarse NiO particles at ~340 °C and the reduction in the large Ni_x_Mg_1−x_O particles at ~505 °C and the small Ni_x_Mg_1−x_O particles at ~660 °C. The decomposition of these large agglomerates leads to the appearance of the hydrogen uptake peak at 360–370 °C, corresponding to the NiO particles only. The same behaviour of the Ni-Mg-O (pH = 5) sample was recently observed using an in situ X-ray diffraction analysis [21].

As follows from Figure 6d, the next sample, Ni-Mg-O (pH = 7), initially consists of a noticeable amount of the large NiO particles (peak at 325 °C) and two types of the Ni_x_Mg_1−x_O particles with the peaks at ~455 °C and ~660 °C. In the subsequent cycles, all three forms are present with the corresponding peaks’ positions at ~350, ~440, and ~530 °C. It should be noted that the peak at ~440 °C gives the main contribution.

The last Ni-Mg-O (pH = 9) sample exhibits two hydrogen uptake peaks at ~350 °C and ~495 °C (Figure 6e). The contribution of the NiO and Ni_x_Mg_1−x_O particles is estimated to be approximately the same. The further reduction behaviour of this sample is similar to the previously described sample Ni-Mg-O (pH = 7). The small Ni_x_Mg_1−x_O particles reproducibly appear in each cycle. Therefore, this sample is characterized by the broad temperature range of the hydrogen uptake, from 290 to 640 °C.

It can be concluded here that the pH of the hydrolysing solution has a strong effect on the strength of the nickel interaction with the MgO matrix. At least four types of nickel species can be considered: the small and large NiO particles non-interacting with the MgO matrix and the Ni_x_Mg_1−x_O species of the NiO-MgO solid solution of large and small sizes with varied content of the incorporated nickel. The contribution of each of these types defines the TPR profile and the hydrogen uptake area.

**Figure 7:**
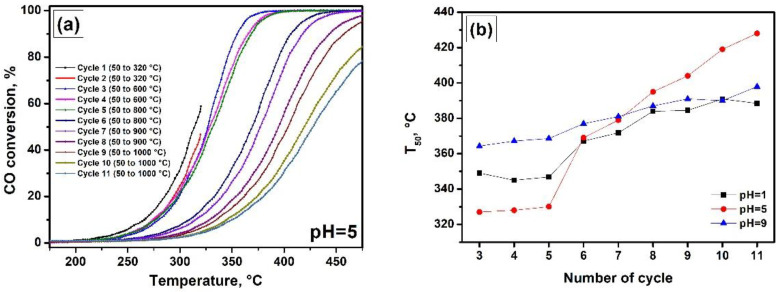
Temperature dependence of CO conversion in a prompt thermal aging regime for the Ni-Mg-O sample obtained at pH = 5 (**a**); thermal stability curves for the Ni-Mg-O samples obtained at various pH (**b**).

Generally, the TPR characterization of the materials is performed along with the temperature-programmed oxidation (TPO) techniques [52,53]. The TPO studies help to reveal the behaviour of the samples under oxidative conditions. In the present research, the CO-TPO method was realized in a prompt thermal aging regime that allowed examining the thermal stability of the Ni-Mg-O samples. This experimental approach is based on the activity of nickel in the CO oxidation reaction. Therefore, this reaction was chosen as a catalytic response. The typical light-off curves of one experiment are shown in Figure 7a. The final temperature of each second catalytic cycle is increased stepwise. Thus, the pairs of curves 2 and 3, 4 and 5, 6 and 7, 8 and 9, 10 and 11 characterize the state of the system treated in the reaction medium at 320, 600, 800, 900, and 1000 °C, correspondingly. Thereby, the shape and position of the light-off curves should not be reproducible. Their shift serves as an indicator of the changed dispersion of nickel species. The monitored parameter was T_50_. Note that in the two first cycles, the temperature of 320 °C was not enough to reach the 50% conversion of CO. Therefore, Figure 7b summarizes the T_50_ values starting from the third cycle.

As evident from Figure 7b, the thermal stability of the Ni-Mg-O system is influenced by the pH. The sample Ni-Mg-O (pH = 5) demonstrates the best initial activity until the aging at 800 °C in the fifth cycle. Such a high thermal treatment results in a sharp increase in T_50_ value, thus testifying towards the agglomeration of the NiO particles. The monotonic deactivation of the sample observed in the subsequent cycles indicates that the agglomeration process continues from cycle to cycle. The Ni-Mg-O (pH = 1) containing small NiO particles and large Ni_x_Mg_1−x_O species, as revealed by the H_2_-TPR method, shows lower initial activity. The aging at 800 °C also negatively affects the catalytic properties of this sample, which is supposedly connected to the enlargement of small NiO particles. However, the observed deactivation is not as crucial as in the previous case. In the further cycles, the activity stabilizes probably due to the presence of the thermally stable Ni_x_Mg_1−x_O species. In contrast to the two described samples, the sample Ni-Mg-O (pH = 9) is less active but most stable. This sample initially contains a smaller amount of dispersed NiO particles along with the dominating number of Ni_x_Mg_1−x_O particles. The high thermal stability of the sample Ni-Mg-O (pH = 9) allows considering it as a promising material for high-temperature exploitation.

## 4. Conclusions

The two-component materials consisting of the MgO matrix, and the transition metal oxide have attracted the attention of researchers for use in chemical looping processes. The present work is devoted to the detailed study of the Ni-Mg-O system prepared by the sol–gel technique. For the hydrolysis of the magnesium methoxide, aqueous solution of nickel nitrate was applied. The effect of pH of the hydrolysing solution was studied for the first time. The desired pH values were obtained by the addition of nitric acid or ammonia. It was shown that these additives decompose with noticeable exothermicity, affecting the textural and morphological properties. Additionally, the pH value at the hydrolysis stage defines the strength of interaction between nickel and the MgO matrix. Thereby, a few nickel-containing species can be formed: dispersed and coarse NiO particles and Ni_x_Mg_1−x_O particles varied in size and nickel content (x). It is important to note that all these forms are relatively small in size if compared with other preparation methods and are uniformly distributed within the MgO matrix. The ratio of these forms defines the properties of the final material. The higher amount of the Ni_x_Mg_1−x_O solid solution species (pH of 7 and 9) makes the system more thermally stable and reproducible in the reduction/oxidation cycles with a wide temperature range of the hydrogen uptake. In contrast, if the NiO particles are the dominant form (pH of 5), the system is less stable and characterized by the narrow hydrogen uptake interval. The samples prepared at acidic pH of 1 and 3 contain both the NiO and Ni_x_Mg_1−x_O particles and take up the middle position.

## Figures and Tables

**Table 1:** Textural characteristics of the Ni-Mg-O samples obtained at various pH.

pH	SSA, m^2^/g	V_pore_, cm^3^/g	D_av_, nm
1	103	1.05	41
3	81	0.67	33
5	154	0.72	19
7	115	0.73	26
9	90	0.83	37

**Table 2:** The lattice parameters (*a*) and estimated crystallite size (D) of the studied samples and NiO and MgO standards.

#	Sample	Lattice Parameter *a*, Å	D, nm
1	Ni-Mg-O (pH = 1)	4.220(1)	7
2	Ni-Mg-O (pH = 5)	4.220(1)	8
3	Ni-Mg-O (pH = 9)	4.219(1)	8
4	NiO (PDF#47-1049)	4.177	-
5	MgO (PDF#45-0946)	4.211	-

## Data Availability

The data presented in this study are available on request from the corresponding author.
